# Later life precarity and longitudinal frailty trajectories in older adults

**DOI:** 10.1017/S0144686X26100543

**Published:** 2026-03-25

**Authors:** Laurence Rowley-Abel, Lisa van den Driest, Qingwen Chen, Yulu Chen, Su Chu, Jessica Lasky-Su, Nicholas J.W. Rattray, Alan Marshall

**Affiliations:** 1 School of Social and Political Science, University of Edinburgh, Edinburgh, UK; 2 Strathclyde Institute of Pharmacy and Biomedical Sciences, University of Strathclyde, Glasgow, UK; 3 Channing Division of Network Medicine, Brigham and Women’s Hospital and Harvard Medical School, Boston, MA, USA; 4 Department of Population Health Sciences, Duke University, Durham, NC, USA

**Keywords:** frailty, inequalities, precarity

## Abstract

The concept of precarity is increasingly being applied in social gerontology to understand risks and uncertainties faced by older adults. However, existing research has not captured precarity quantitatively nor has it modelled its effect on older adults’ health. We therefore develop a Later Life Precarity Index and model its association with frailty. Using longitudinal data on 15,733 older adults from the English Longitudinal Study of Ageing, we examine the association between frailty and risks across the domains of finances, pensions, employment, housing, relationship and unpaid care-giving. We then develop the Later Life Precarity Index and model its longitudinal association with a Frailty Index using hybrid panel regression. The results indicate that multiple social risks are strongly and independently associated with frailty, particularly around finances, housing, fuel poverty and food insecurity. In longitudinal models, the precarity index explains both between-individual differences and within-individual changes in frailty and performs substantially better than standard measures of socio-economic status (wealth and education). The strong longitudinal association of the precarity index with frailty suggests that social gerontology’s growing focus on precarity is a useful lens for understanding the diverse, changing and new forms of social risk that impact frailty. By developing, testing and sharing this novel measure of later life precarity, this study brings potential for new understandings of the evolving drivers of inequalities in the health of older adults.

## Introduction

The concept of precarity is increasingly being applied in the field of social gerontology to examine the exposure to diverse forms of risk and uncertainty that are a part of the ageing process (Grenier et al. 2019; 2020). Unlike other concepts used to examine social inequalities in older adults, such as socio-economic status, precarity has a focus on insecurity and a generalised vulnerability to external circumstances. The concept has largely been developed in research on the structural changes in employment conditions in recent decades that have seen the growth of unstable, insecure forms of work (Standing 2011). This employment-focused notion of precarity has been applied in social gerontology to examine the effects of unstable employment trajectories and retirement transitions amongst older adults (Raymo et al. 2011; Wahrendorf et al. 2020). However, precarity has also been used to understand growing exposure to risk in other social domains. For example, in the sphere of housing, the lens of precarity has been used to highlight the growth in the proportion of older adults who continue to rent during later life and how this exposes them to a state of uncertainty that interacts with other forms of risk, such as health and care-giving (Bates et al. 2019; Age UK 2023; Petersen and Tilse 2023). Similar forms of exposure to uncertainty have been highlighted in spheres such as social care, where the crisis in formal social care services has led to increasing reliance of older adults on informal, precarious forms of care-giving (Burchardt et al. 2016). As such, precarity does not just represent insecurity faced by older adults in a single domain; rather, it represents a generalised exposure to risk across multiple domains that results in ‘life worlds that are inflected with uncertainty and instability’ (Waite 2009, p. 416) and which arise out of policy contexts shaped by a retrenchment of the welfare state, rearrangement of labour market conditions and a decline in state-provided services (Phillipson 2020).

However, in existing research, few studies have attempted to quantitatively capture precarity as it pertains to older adults. While certain studies have quantitatively examined individual dimensions of precarity, such as employment insecurity (*e.g*. Raymo et al. 2011; Wahrendorf et al. 2020), to our knowledge, none have attempted to capture the generalised exposure to risk across multiple domains that precarity represents. Equally, existing studies have not quantitatively modelled the association between later life precarity and health in older adults. Again, individual dimensions of precarity have been studied – such as the effects of housing insecurity on biological ageing (Clair et al. 2023) or of care-giving on physical and mental health (Brimblecombe and Cartagena Farias 2022) – but the effect of overall precarity has not been examined.

In this study, we therefore develop a measure of later life precarity and examine its association with frailty in older adults. Frailty represents a state of generalised physiological vulnerability and loss of resilience that increases as individuals age. It is a particularly salient health outcome to study in relation to precarity, given the fact that it captures a multi-dimensional risk state that is focused on dysregulation across multiple biological systems (Theou et al. 2015) and as such mirrors precarity’s focus on risk and vulnerability across *multiple* social domains. Frailty is also a key metric of healthy ageing and is strongly associated with the risk of mortality, hospitalization and disability (Vermeiren et al. 2016). Grenier (2020) has explicitly linked frailty and precarity, arguing that precarity offers a framework through which we can better understand the frail state as one that arises in a social and political context marked by features such as the marketisation of formal care services, rather than it being purely a biomedical product. Here, we build on this argument by explicitly modelling the association between frailty and the social risks faced by older adults in such precarious social and political contexts. There is also a substantial literature demonstrating the inequalities in frailty prevalence by socio-economic status (*e.g*. Marshall et al. 2015; Brunner et al. 2018; Uccheddu et al. 2019). Using the lens of precarity allows us to add to this literature by focusing on the distinct risks (such as poor housing conditions or food insecurity) that may contribute to these inequalities, rather than focusing on markers of status itself (such as education or occupational class). This is of particular importance for developing policy interventions that seek to alleviate these inequalities.

This study, therefore, develops a measure of later life precarity that captures the diverse forms of social risk faced by older adults and tests this measure’s longitudinal association with frailty. Specifically, we examine the association between frailty and precarity relating to finances, pensions, employment, housing, relationships and care-giving. Below, we briefly set out our rationale for focusing on these six domains of precarity, before setting out the data and methods used in the analysis and then presenting and discussing our findings.

## Domains of precarity

Discussions of precarity in the field of social gerontology have highlighted numerous ways in which older adults are exposed to social risks and uncertainty as they age. While such discussions have covered a wide range of areas, there are several key themes that often emerge: finances, pensions, employment, housing, relationships and care-giving. When developing our measure of later life precarity, we therefore focused on risks from each of these six domains. In this section, we briefly outline how each of these themes relates to precarity in the context of later life.

### Finances

Uncertainty and vulnerability relating to finances has been a key theme in the literature on precarity. In particular, there has been a focus on how insecure work and the rearrangement of traditional employment conditions have eroded stable sources of income for many individuals (Standing 2011). In the context of later life, Marshall et al. (2024) highlight the precarity of income trajectories faced by many older adults, particularly around the transition into retirement. Notably, they demonstrate that the level of income precarity is determined not only by employment-related factors but also by factors such as martial status, care-giving responsibilities and housing tenure. Additionally, the retrenchment of the welfare state in many contexts has meant that older adults’ access to sources of income outside of employment (*e.g*. health-related benefits) is becoming increasingly restricted and uncertain, further contributing to financial precarity when poor health or care-giving interferes with older adults’ ability to undertake paid employment (Lain et al. 2018).

### Pensions

While strongly related to finances, pensions access represents a unique financial risk that is specific to later life and determined by a complex set of factors that go beyond finances, such as gender and marital status (Buckley and Price 2021). Standing (2011) points to pensions insecurity as a key element contributing to growing precarity. He argues that a decline in employers’ commitment and contribution to workers’ pensions has reduced the value of occupational pensions, and is part of a more general shift away from employers providing security to their employees. Equally, Lain et al. (2018) highlight how the deferral of the age at which individuals can access a state pension engenders further precarity amongst older adults by removing a social safety net that supports the transition out of work.

### Employment

The growth of insecure forms of employment is a central focus of precarity (Standing 2011). In the context of various policy changes (*e.g*. deferral of the state pension age), shifts away from ‘lifelong’ jobs and the effects of the post-2008 recession and austerity, older individuals have longer working lives than in previous decades but also experience more precarious attachment to the labour market (Parsons and Walsh 2019). As a result, ‘many older people are being forced to re-start or re-imagine their working lives at a time when many would have been expecting to enjoy peak earnings and secure employment’ (Parsons and Walsh 2019, p. 12). This is further complicated by insecurities around retirement, with poor jobs meaning individuals may have to delay retirement due to limited savings, or precarious attachment to the labour market meaning individuals may become retired involuntarily. This contributes to the move from employment to retirement being a particularly precarious lifecourse transition (McGann et al. 2016; Settersten 2020).

### Housing

Increasing attention is being paid to how poor-quality or insecure housing creates precarious ageing environments. Decreases in home ownership mean that a growing proportion of older adults are continuing to rent in later life (Age UK 2023). Older tenants face insecurities around the possibility of eviction, substantial proportions of income being absorbed by rent costs, uncertainty around rent increases and difficulty securing home adaptations required for their care needs (Bates et al. 2019; Storey and Coombs 2020; Petersen and Tilse 2023; Brimblecombe et al. 2025). On top of this insecurity, older renters are also more likely than home owners to live in poor-quality housing with problems such as damp and cold (Age UK 2023). At its extreme, housing precarity can result in homelessness in later life, which may be triggered by events such as leaving work due to poor health or relationship dissolution (Bates et al. 2019; Petersen and Tilse 2023).

### Relationships

Events such as widowhood, divorce and separation in later life can lead to older adults experiencing precarious and isolated circumstances. For women in particular, such events can lead to especially precarious financial situations due to their reliance on a spouse’s income, stemming from gendered inequalities in pensions access, wages and care-giving responsibilities (Buckley and Price 2021). Marshall et al. (2024) have also shown how widowhood and divorce are linked to more precarious income trajectories during later life. At the same time, relationship dissolution is often a precursor to older adults’ experiencing housing precarity and leads to individuals having to leave the homes in which they were expecting to age (Bates et al. 2019). Similarly, living alone has been highlighted as a factor associated with an especially precarious experience of ageing – Portacolone et al. (2018, p. 272) show how older adults living alone experience precarity as ‘an intrinsic sense of uncertainty resulting from coping with cumulative pressures while trying to preserve a sense of independence’, particularly in contexts where their desire for independent living is only poorly supported by external public services that are insufficient or hard to access.

### Unpaid care-giving

The crisis in formal social care provision means that large quantities of care to older adults are provided in an informal capacity by relatives or friends (The King’s Fund and Nuffield Trust 2016). Fine (2020) argues that this reliance on informal, unpaid care is a form of precarity in the sense that it exposes individuals to being contingent on unstable and irregular sources of support. Fine also highlights how, due to insufficient provision by public and private care systems, unpaid care-giving has emerged as a further example of precarious work that many older adults must undertake. Similarly, Burchardt et al. (2016) highlight the precarious position of unpaid carers in the context of cuts to formal care services, with the uncertainty and insufficiency of publicly funded services meaning they have to change working patterns, leave employment or move home to take on greater amounts of care-giving when services are withdrawn.

## Methods

### Dataset and analytic samples

Our study uses data from the English Longitudinal Study of Ageing (ELSA, www.elsaproject.ac.uk) (Steptoe et al. 2012), a large, nationally representative panel study of adults aged 50 and over living in England. We use both the original ELSA dataset and the harmonised version of the dataset produced by the Gateway to Global Aging (Wilkens et al. 2023). In all, 11,391 core participants were first interviewed in 2002/03 and have been followed up every two years subsequently. Refreshment samples have been added at later waves to keep the sample representative of the population aged 50 and over. We use data from Waves 2–9, conducted from 2004/05 to 2018/19. These waves were selected as data on certain social factors of interest was not available in Wave 1, and harmonised data was not yet available beyond Wave 9.

In total, 17,722 individuals were interviewed across Waves 2–9, resulting in 77,970 observations. Individuals with at least one valid observation across the relevant waves were included in our analysis. We excluded 991 individuals aged under 50 – these individuals are partners of core sample members or are other household members and are therefore in ELSA despite being under 50. Our measure of frailty was based on 56 variables (further details below) and we excluded 27 individuals who had fewer than 30 of these variables with non-missing values, following standard practice (Searle et al. 2008). We also excluded individuals with missing data for any of the social risks included in the measure of precarity, which resulted in a sample size of 15,733 individuals with 62,694 observations for the main models. When comparing this analytic sample with the full sample at baseline, no significant differences were found in terms of age, sex, frailty, wealth or income (see Supplementary Table 1). Additionally, sensitivity analyses using longitudinal inverse probability weighting to account for non-random missingness produced substantively similar results (further details below).

### Measuring frailty

To measure frailty, we used a Frailty Index (FI) that has previously been established in ELSA (Marshall et al. 2015), but updated it to reflect changes in the data being collected in more recent waves. The index considers 56 deficits across six domains – physical function, cognitive function, activities of daily living, chronic conditions, psychological health and general health. We calculated the FI in the way set out by Searle et al. (2008), scoring a deficit as either present (1) or absent (0) and calculating the mean value across all deficits for an individual (*i.e*. the proportion of deficits present). For some deficits, such as self-rated eyesight, a graded score is given rather than a binary indicator (*e.g*. 0 = excellent eyesight, 0.2 = very good eyesight etc.). Details of the full set of deficits and their scoring can be found in Supplementary Table 2. We calculated the FI at each of the eight waves used in our analysis.

### Modelling individual social risks and frailty

In the first stage of the analysis, we identified social risks that may contribute to precarity and tested whether they were associated with frailty. To do this, we initially selected 31 candidate social variables from the domains of finances, pensions, employment, housing, relationships and unpaid care-giving. The full set of candidate variables is provided in Table 1. For risks relating to employment, such as job loss, measures referred to whether the respondent had ever experienced such an event either at the current or previous ELSA waves (or as reported in the life-history module for certain variables). This was because very frail individuals are less likely to be currently in the labour market, but adverse employment events may have affected their health previously. For similar reasons, risks relating to care-giving also referred to whether the respondent had ever reported such risks at the current or previous ELSA waves. Likewise, the homelessness variable referred to homelessness experienced prior to ELSA, as currently homeless individuals would not be in the study.

To test which risks were associated with frailty, we ran linear random-intercept mixed-effect models (specifically, random-effects panel regression) of the FI, with each factor entered as an independent variable one at a time. The models also controlled for age, age^2^ and sex. All variables that were statistically significant (p < 0.05) and positively associated with frailty were then selected for further analysis. The 21 selected variables are marked (†) in Table 1. We then considered whether these 21 social factors were associated with frailty independently of each other by entering all of the variables into a model simultaneously, along with age, age^2^ and sex (Model 1). Given that some of the 21 factors may be strongly correlated, we also ran sensitivity analyses to identify multi-collinearity issues (see below for further detail). Robust standard errors were used for all models since the residuals from initial models were skewed.

### Deriving the Later Life Precarity Index

In the second stage of the analysis, using the 21 variables previously selected, we derived a composite measure of social risks associated with frailty, which we term a Later Life Precarity Index (LLPI). We derived the LLPI using the baseline (*i.e*. Wave 2) observations. To do this, we used elastic net regression (Zou and Hastie 2005) to regress the FI on the 21 social variables, quadratic terms of all continuous variables and all two-way interactions. Optimal values of lambda and alpha were selected through cross-validation. We included quadratic terms as some social determinants have been shown to have a non-linear relationship with frailty (*e.g*. Brunner et al. 2018). Interaction terms were included since a key element of precarity is how insecurities can intersect and exacerbate each other’s effects (Grenier et al. 2019). Having trained the elastic net model, the regression weights were then used to produce the LLPI (this is akin to using the model coefficients to produce predicted outcome values in ordinary least squares regression). The resultant index ranged from −0.020 to 0.480. We examined the association of the LLPI with the FI cross-sectionally using a scatter plot of the two variables and calculating their R^2^.

### Modelling the longitudinal association between the Later Life Precarity Index and frailty

Having derived the LLPI, in the final stage of the analysis, we looked at its longitudinal association with the FI. We calculated the LLPI at each wave (using the same weights as derived in the previous analysis) and then ran hybrid panel regression models (Allison 2009). This is a type of mixed-effects model that estimates both the effect of the LLPI on within-person fluctuations in frailty (the within-effect) and the effect on between-person differences in frailty (the between-effect). This model was selected as it provides estimates of the within-effect while also allowing for time-constant independent variables. Testing the within-effects directly provides more robust support for a causal relationship between precarity and frailty, since unobserved time-constant variables are controlled for as individuals act as their own control. We started with a base model containing only age, age^2^ and sex (Model 2), and then added the LLPI (Model 3). To test how the LLPI compared to standard markers commonly used to control for socio-economic circumstances, we also ran a model with wealth percentile and education (Model 4). All of the continuous independent variables were z-transformed for ease of interpretation. Robust standard errors were used in the models due to skewed residuals.

### Sensitivity analyses

In the panel regression model containing all of the social risks as independent variables simultaneously (Model 1), there is a risk that strong correlations between the independent variables could introduce issues of multi-collinearity. To test for this, we ran a sensitivity analysis where highly correlated social risks were removed from the model.

Additionally, all of the models use unweighted data in order to maximise the sample size, since longitudinal weights are only available for individuals who responded to all waves of ELSA. It was important to maximise the sample size, since some elements of precarity (*e.g*. homelessness) are relatively rare. However, unweighted estimates may not be representative of the population, especially due to bias introduced through sample attrition across waves. We therefore ran versions of our main models (Models 2–4) using longitudinal inverse probability weighting as a further sensitivity analysis. A weighted version of Model 1 was not run as the substantially reduced sample size meant that many of the social risk variables had few individuals experiencing the risk.

Finally, in the hybrid panel regression with wealth and education as independent variables (Model 4), wealth was entered as a continuous variable measured in percentiles for the sake of model parsimony. However, conventionally, wealth is often measured in quintiles and entered as a categorical variable in order to allow for non-linear effects. To test if this operationalization of wealth altered our findings, we ran a sensitivity analysis where Model 4 was re-run using a categorical wealth quintile variable instead of the continuous wealth percentile variable.

### Ethics

The ELSA study team obtained written, informed consent from all respondents for their participation in the study. All analyses were conducted following the end user licence of the data providers and the UK Data Service.

## Results

Table 2 presents the baseline characteristics of the sample. The mean age was 62.17 and women made up 54.34 per cent of the sample, consistent with the slightly greater proportion of women than men amongst the older adult population in England. The mean FI was 0.14. For reference, values of 0.1 and 0.25 are commonly used thresholds for defining pre-frailty and frailty, respectively, although there is considerable variation between studies (Gordon et al. 2021). The median total wealth was £200,600, while the median total annual income was £19,866.

### Testing individual social risks and frailty

We first tested whether each of the social risks (listed in Table 1) was longitudinally associated with the FI using linear random-intercept mixed-effects models (specifically, random-effects panel regression), with one factor modelled at a time. The models also controlled for age, age^2^ and sex. Variables that were statistically significant (p < 0.05) and positively associated with frailty were then selected, resulting in 21 variables to be used in further analysis. The 21 selected factors are marked (†) in Table 1 (full model results are provided in Supplementary Table 3).

**Figure 1:**
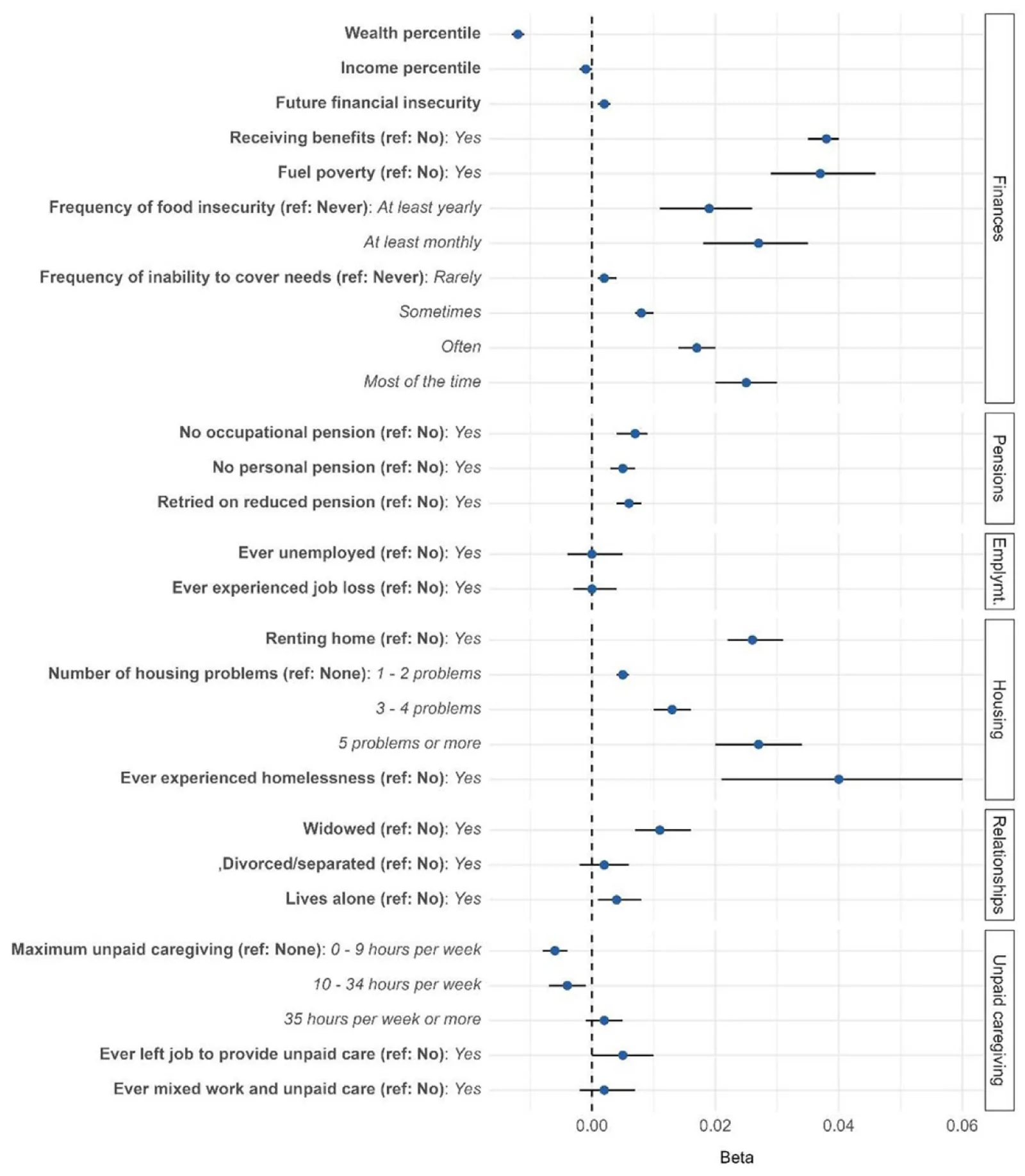
Coefficients for Model 1. *Notes:* Random-effects panel regression model of frailty, with social risk variables, age, age^2^ and sex as independent variables. N observations = 62,694. N individuals = 15,733.

We then modelled the 21 selected variables simultaneously, to test their association with the FI independent of each other, controlling for age, age^2^ and sex (Model 1). Figure 1 presents the model coefficients (full results are provided in Supplementary Table 4).

All of the financial risks were associated with worse frailty. Beyond the standard financial measures of wealth, income and benefit receipt, factors relating to material deprivation (food insecurity and fuel poverty) were independently and strongly associated with frailty. Future financial insecurity (*i.e*. self-reported concerns about future finances) was also associated with frailty, independent of an individual’s current financial circumstances. Each of the pensions variables showed small but significant associations with frailty, while neither of the employment variables showed significant effects. All of the housing variables – renting, housing problems and homelessness – showed substantial effects on frailty. In terms of relationships, being widowed and living alone were associated with small elevations in frailty, while being divorced had no significant effect. Compared to those who had never provided unpaid care, having ever provided relatively low amounts of unpaid care was associated with reduced frailty. However, having provided intensive levels of unpaid care showed no significant effect. Having left a job to care was associated with slightly worse frailty, while having mixed work and care was not significant.

### Deriving the Later Life Precarity Index

**Figure 2:**
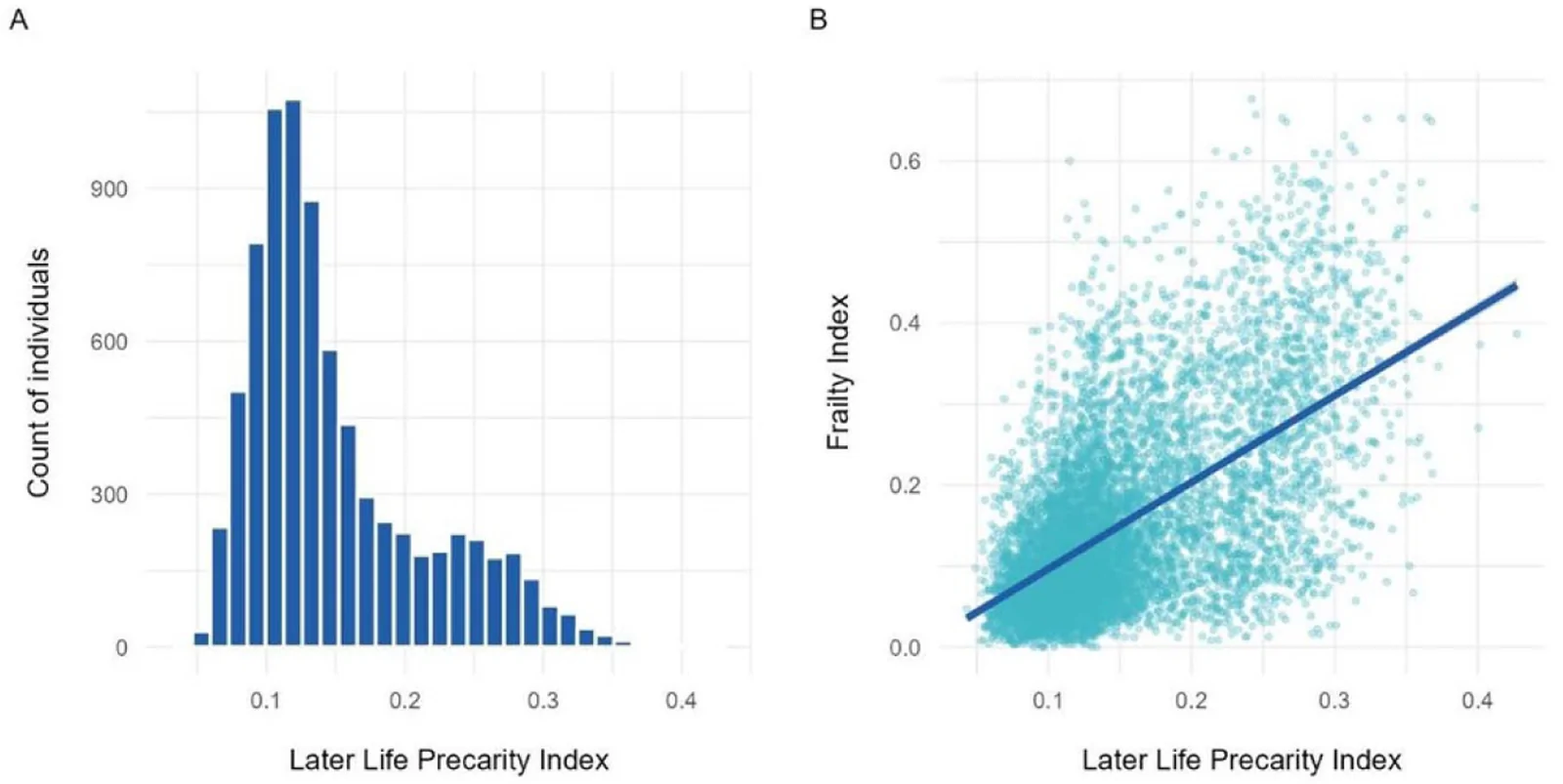
Histogram of LLPI and scatter plot of FI and LLPI. *Notes:* A) Histogram of Later Life Precarity Index at baseline (Wave 2). B) Scatter plot of Frailty Index and Later Life Precarity Index at baseline (Wave 2).

Having examined these individual social risks, we then derived a composite index of overall precarity that captured social risk associated with frailty – the Later Life Precarity Index (LLPI). This was done by regressing frailty on the selected social risk variables in the baseline data using elastic net regression and using the resultant model weights to derive the index (see Methods for further details). Figure 2a shows the distribution of the LLPI, while Figure 2b shows its association with the FI. The R^2^ between the LLPI and the FI was 0.350 (p < 0.001).

### Testing the longitudinal association between the Later Life Precarity Index and frailty

We then calculated the LLPI at all waves in our dataset, such that we now had time-varying measures of precarity and frailty. Using hybrid panel regression, we modelled the longitudinal association between the LLPI and the FI. Table 3 presents results for Models 2–4. The LLPI, age and wealth variables have been z-transformed to facilitate interpretation of the coefficients.

The LLPI was strongly and statistically significantly associated with both between-person differences and within-person changes in frailty. A one standard deviation difference in the LLPI between individuals was associated with a difference of 0.068 on the FI (p < 0.001). To put this in context, a 0.068 increase in the FI is approximately equivalent to the increase in frailty experienced by the average woman between the ages of 50 and 80, according to predicted values from Model 2 (see Supplementary Materials Appendix 1 for details on the calculation method). A one standard deviation increase in the LLPI within an individual over time was associated with an increase in the FI of 0.012 (p < 0.001). This increase is approximately equivalent to the increase in frailty experienced by the average woman between the ages of 50 and 63. Additionally, there is a notable decrease in the coefficient for sex, between Model 2 (b = 0.019, p < 0.001) and Model 3 (b = 0.004, p = 0.002). This decrease of 78.9 per cent in the sex coefficient suggests that the elements of precarity included in the LLPI may account for a large proportion of the sex-differences observed in frailty, though further research is required to confirm and explore this result.

The model with the LLPI also explained more of the variation in the FI than the model containing only age, age^2^ and sex. In terms of explaining within-person change in frailty, the LLPI provided a small improvement to the model fit (R^2^ increased from 0.134 to 0.147). In terms of explaining between-person differences in frailty, the LLPI added substantial explanatory power (R^2^ increased from 0.109 to 0.426). The model with the LLPI also explained substantially more variation in the FI than the model with wealth and education, with the overall R^2^ being 0.393 for Model 3, but only 0.228 for Model 4. Additionally, the effect size for the LLPI was substantially largely than that of wealth or education.

### Sensitivity analyses

In Model 1, which contained all the selected social risk variables simultaneously, we tested for multi-collinearity issues by removing highly correlated variables. Two pairs of variables displayed correlations over 0.7 – renting and wealth percentile, and the maximum amount of unpaid care provided and having ever mixed work and care. In the sensitivity analysis model, we removed the variables renting and having ever mixed work and care. This model provided substantively the same results, except that the coefficient for the highest category of unpaid care-giving became marginally statistically significant (see Supplementary Material Figure 1).

Additionally, the models presented above were unweighted in order to maximise the sample size, since longitudinal weights are only available for individuals responding to all waves. To check the effect of weighting, we ran weighted versions of our main models (Models 2–4). These produced substantively similar results to the unweighted models, in that the LLPI was strongly and significantly associated with both within- and between-variation in frailty and performed better than wealth and education at explaining frailty. Results of the weighted models can be found in Supplementary Table 5.

As a final sensitivity analysis, for Model 4, we checked whether operationalizing wealth as a categorical variable measured in quintiles (rather than as a continuous variable measured in percentiles) changed our results substantively. In this model, there were slight non-linear effects, with the difference between the poorest quintile and the second poorest quintile being larger than the difference between subsequent quintiles. However, the results were substantively the same and the model fit remained similar (overall R^2^ = 0.225 and 0.228 for the model with quintiles and the model with percentiles, respectively). Therefore, our finding that the LLPI explained variation in frailty better than standard socio-economic markers remained the same. Full model results are provided in Supplementary Table 6.

## Discussion

Several key findings emerge from these results. Firstly, a diverse set of vulnerabilities across various domains of precarity are associated with frailty independently of each other. Secondly, an overall measure of precarity explains a substantial proportion of the variation in frailty and is able to explain both between-person differences in frailty and within-person changes in frailty over time. Thirdly, a large amount of the sex-differences observed in frailty appear to be explained by our measure of precarity. Finally, exposure to precarity explains frailty better than standard markers of socioeconomic status (wealth and education). Below we discuss each of these findings in more detail.

Firstly, our analysis identifies a number of elements of precarity that are associated with worse frailty. As well as low income and wealth, the variables indicating renting in later life, food insecurity, fuel poverty, homelessness and poor housing quality stand out as risks that have particularly substantial effects. There are also smaller, but nonetheless statistically significant, effects of exposure to risks in the areas of pensions, relationships and unpaid care-giving. It is notable that, despite the large number of closely related variables included in the model, many of the risks show independent associations with frailty. This suggests that exposure to insecurity across many social domains contributes to the development of frailty, which is consistent with precarity’s conception of a generalised and multi-dimensional vulnerability. Our models suggest that risks accumulate across the domains of finances, pensions, housing, relationships and unpaid care-giving, creating lives characterised by precarity in many dimensions, and that these exposures have independent effects on the ageing process.

It is also notable that several of the elements of precarity with the strongest effects are related to housing. This result highlights housing as a domain that can be particularly important for determining the course of ageing with implications for potential policy interventions around specific housing precarities. The importance of housingrelated risks in our models is also consistent with a study which found that renting and poor housing quality are substantially associated with faster biological ageing (measured using DNA-methylation) (Clair et al. 2023). In the context of increasing housing insecurity amongst older adults and a growing proportion of individuals continuing to rent during later life, these substantial detrimental effects are particularly concerning and further underline the need for housing policy to facilitate individuals to own their own home, provide greater security and rights for those who are renting, and ensure that housing is of a proper standard.

It should be noted that while it may seem unexpected that individuals who had provided low amounts of unpaid care had lower frailty (compared to those who had never been a care-giver), this finding is in line with previous research that has found that providing smaller amounts of care is associated with better health, while providing large amounts of care is associated with worse health (Vlachantoni et al. 2013). Our finding may be due to the fact that those who had been in poor health, even at the beginning of the ELSA study, might never have been able to provide care for someone else due to their own health issues. Equally, the large number of other risks included in the model may mean that the negative effect of care-giving is being captured through the other variables, such as having to leave work to provide care and the variables measuring poor financial circumstances. This is supported by the fact that when removing strongly correlated variables in the sensitivity analysis, the highest level of care-giving became statistically significant and positively associated with frailty. On the other hand, lower amounts of care-giving may be genuinely having a positive impact on frailty – previous research has shown links between low-level caring and volunteering, and there is some evidence that, in this way, small amounts of care-giving may have positive effects on health (Burr et al. 2005; O’Reilly et al. 2017). For this reason, low-intensity care-giving is often seen as a factor that can potentially play a part in fostering ‘productive ageing’, a paradigm focused on the positive contributions made by older adults to wider society which bring benefits to both older adults themselves (*e.g*. social connection) and their communities (*e.g*. providing childcare) (Gonzales et al. 2015). Nonetheless, the connection between care-giving and greater wellbeing is complex and dependent on a range of contextual factors (Vlachantoni et al. 2013).

Secondly, our measure of precarity – the LLPI – accounts for a substantial proportion of the variance in frailty. In longitudinal models, we find that the effect size of the LLPI is large – a one standard deviation difference between individuals is associated with a change equivalent to the increase in frailty experienced by the average woman between the age of 50 and 80. Additionally, we find not only that more precarious individuals tend to be frailer compared to less precarious individuals but also that if someone’s circumstances worsen over time, they also become frailer, above and beyond the effect of chronological ageing. This significant within-person effect is consistent with the idea that there is a causal relationship between precarity and frailty. It is also notable since many existing studies that focus on *biological* mechanisms behind ageing and frailty have found evidence of cross-sectional between-person effects, but limited or no evidence of longitudinal, within-person effects (Gonçalves et al. 2022). For example, Marioni et al. (2015) find that epigenetic age acceleration is associated with baseline physical function, but not change in physical function over a six-year period. More recently, while Mak et al. (2023) found that five different biological clocks were able to predict baseline frailty, only one (DunedinPACE) was able to predict change in frailty over time. We highlight this not to suggest that epigenetic or other biological factors are not important in the ageing process but rather to show that, if we want to understand how frailty develops over time within an individual, we need a detailed understanding of changes in the social factors to which they are exposed as well as the biological ones. While biomarkers may act as useful signals of an accelerated ageing process, analysing social factors, as we do here, is important for capturing the underlying drivers behind these signals.

Thirdly, our models are consistent with a hypothesis that worse frailty amongst women compared to men is explained to a large extent through women being exposed to greater precarity in later life than men. This finding is noteworthy since, although the existing literature has investigated whether the predictors of frailty are different in women and men (Alexandre et al. 2018; Oberndorfer et al. 2021; Mielke et al. 2022), there has been little investigation of what factors explain the sex-difference in frailty itself. Hubbard and Rockwood (2011) proposed that greater physiological reserve amongst women may allow them to survive longer with a higher level of frailty than men and also suggest that differences in social vulnerability may be important. Our findings suggest that social vulnerability – conceived here as precarity – is indeed a very significant factor in explaining why women experience worse frailty than men. While we do not formally test the mediating effect of precarity between sex and frailty, this result around sex-differences in frailty and the LLPI fits with existing literature that has shown that, compared to men, older women are exposed to more precarious circumstances in later life across areas such as insecure finances (Price 2006) and unpaid care-giving (Brimblecombe and Cartagena Farias 2022), and that they also face higher rates of frailty (Hubbard and Rockwood 2011). This finding points to a promising avenue for future research seeking to understand the mechanisms behind these sex-differences.

Finally, we found that precarity explains frailty better than standard markers of socio-economic status, such as wealth and education. This suggests that precarity is a useful lens to use when analysing inequalities in the ageing process and how it is changing over time as experiences and circumstances of the older population become increasingly diverse. Precarity goes beyond the focus on markers of social position and instead focuses on the distinct, material risks faced by older adults with low socio-economic status, and our findings suggest that this focus provides meaningful insights into the factors driving the social gradient in ageing outcomes. As such, when modelling inequalities in the health of older adults, simple markers of social position are not sufficient to capture the wide array of risks that could be having an impact. Equally, studies that seek to control for social determinants of frailty alongside their main exposure of interest may need to look beyond standard variables such as wealth and education and include more detailed variables in order to account for the diverse forms of precarity that older adults now face. More broadly, cohorts of older adults are increasingly experiencing ageing in the context of numerous crises, be that austerity in the aftermath of the 2008 financial crisis, the social care crisis, the Covid-19 pandemic or the cost-of-living crisis. For these cohorts, the ageing process therefore occurs within an environment characterised by multi-dimensional risks, and indeed previous research has linked contexts such as post-2008 austerity with worse ageing outcomes (Pugh et al. 2024). Our findings suggest that precarity is a useful way of understanding these adverse social environments and the numerous exposures that come with them, and it is better able to capture these vulnerabilities than standard markers of socio-economic status.

Certain limitations of the study should be considered when interpreting these findings. Firstly, despite using longitudinal models that attempt to minimise the bias due to unobserved variables, our findings cannot be taken as proof of a causal relationship between precarity and frailty and nor do we claim this. For example, reverse causality may be occurring whereby becoming frail leads to higher likelihood of experiencing certain social risks (*e.g*. frail individuals starting to receive health-related benefits). However, to some extent, these issues are inherent in the observational nature of the study and our use of hybrid panel regression models aims to mitigate the risks of detecting spurious relationships by considering both within-person and between-person effects. Secondly, sample attrition between waves also introduces the potential for bias, since frailer and more socially vulnerable individuals will have a higher probability to drop out of the sample (*e.g*. due to higher mortality amongst these groups). However, sensitivity analyses using weighted data produced substantively similar results (see Supplementary Table 5 for results of weighted analyses).

Code to derive the Later Life Precarity Index, as well as the detailed social risk variables that go into it, has been made publicly available in the following GitHub repository: https://github.com/lrowleyabel/Precarity-and-Frailty. Future research may draw on the index and its constituent risks to further investigate precarity in later life, as well as social inequalities in ageing more broadly. For example, research examining ethnic, sex or geographic inequalities in ageing might benefit from examining how precarity intersects with each of these domains. Equally, studies seeking to explore phenomena such as the levelling-off in life expectancy that has occurred in the past decade (Darlington-Pollock et al. 2021) might investigate how levels of precarity differ across cohorts of older adults.

Overall, our results show that a substantial association exists between frailty and precarity in older adults. Beyond traditional financial measures, we demonstrate that various elements of precarity are associated with frailty, particularly around housing, food insecurity and fuel poverty. Our measure of precarity is able to track both between-person differences and within-person fluctuations and explains frailty better than standard markers of socio-economic status. This suggests that the more holistic lens of precarity, with its focus on distinct material risks, provides meaningful insights into the mechanisms driving inequalities in ageing. Our analysis shows how vulnerabilities across various domains impinge on health in later life – older adults face adverse exposures, ranging from unstable finances, lack of access to a pension, poor housing quality or difficulties heating their homes, and these risks can then accumulate to produce a precarious ageing environment. As we contend with a rapidly ageing population, adequate policies that address these specific risks and reverse trends such as the growth of renting amongst older adults are needed in order to ensure that later life is not a period that for many becomes characterised by frailty, vulnerability and precarity.

## Figures and Tables

**Table 1:** Details of candidate social risk variables

Domain	Social factors	Description
Finances	Wealth percentile^†^	Percentile of net worth of savings/debt, including financial assets and property, measured at benefit-unit level
	Income percentile^†^	Percentile of total income excluding benefits, measured at benefit-unit level and equivalised for benefit-unitsize
	Benefit receipt^†^	Whether receiving benefits (yes/no)
	Frequency of inability to cover needs^†^	How often does not have enough money to cover needs of household (rarely/sometimes/often/most of the time)
	Future financial insecurity^†^	Self-reported likelihood of not having enough money to cover needs in future (0 = absolutely no chance to 100 = absolutely certain)
	Fuel poverty^†^	Spending more than 10 per cent of income on energy costs (yes/no)
	Food insecurity^†^	How often anyone in the household has skipped meals or cut the size of meals due to lack of money (never/at least yearly/at least monthly)
Pensions	Lack of personal pension^†^	Does not have a personal private pension, regardless of whether or not currently contributing or receiving (yes/no)
	Lack of occupational pension^†^	Does not have an occupational private pension, regardless of whether or not currently contributing or receiving (yes/no)
	Retired on a reduced pension^†^	Had a reduced pension or no pension when first retired due to early retirement (yes/no)
Employment	Ever unemployed^†^	Reports having been unemployed currently, at previous waves or prior to the first wave (yes/no)
	Ever experienced job loss^†^	Reports having lost a job (due to redundancy, dismissal, company closure etc.) currently, at previous waves or prior to the first wave (yes/no)
	Ever worked second job	Reports having worked a second job either currently or at previous waves (yes/no)
	Ever worked part-time	Reports having worked part-time either currently or at previous waves (yes/no)
	Ever self-employed	Reports having been self-employed either currently or at previous waves (yes/no)
	Ever agency worker	Reports having been an agency worker either currently or at previous waves (yes/no)
	Ever worked on fixed-term contract	Reports having been employed on a fixed-term contract either currently or at previous waves (yes/no)
	Involuntary retirement	Whether retired involuntarily, due to redundancy, dismissal, company closure etc. (yes/no)
Housing	Renting home^†^	Rents home (yes/no)
	Number of housing problems^†^	Number of problems *(e.g.* damp, pests, noise) reported with housing (None/1−2 problems/3−4 problems/5 problems or more)
	Ever experienced homeless-ness^†^	Reports ever having been homeless for one month or more in the life-history module (yes/no)
	Paying mortgage	Whether paying a mortgage (yes/no)
	Overcrowded housing	Whether housing has fewer than the standard number of rooms for the household (yes/no)
	No central heating	Whether housing has no central heating (yes/no)
	Involuntarily moved home	Whether moved home involuntarily in the past five years due to eviction etc. (yes/no)
Relationships	Living alone^†^	Lives as the only person in the household (yes/no)
	Widowed^†^	Reports marital status as widowed (yes/no)
	Divorced/separated^†^	Reports marital status as divorced/separated (yes/no)
Unpaid care-giving	Maximum unpaid care-giving^†^	Maximum amount of unpaid care-giving, at current wave or previous waves (None/0−9 hours/10−34 hours/35 hours or more)
	Ever mixed employment and unpaid care-giving^†^	Ever reported combined working and care-giving hours being at least 35 hours per week, with at least 7 hours from working and 7 hours from care-giving, either currently or at previous waves (yes/no)
	Ever left job to care^†^	Ever left job to care for ill or disabled relative or friend (yes/no)

† Variable is positively and statistically significantly associated with frailty in initial models.

**Table 2:** Baseline characteristics of main analytic sample

Age	*Minimum*	50
	*Maximum*	90
	*Median*	60
	*Mean*	62.17
	*Standard deviation*	9.82
**Sex**	*Male (%)*	45.66
	*Female (%)*	54.34
**Frailty Index**	*Minimum*	0.00
	*Maximum*	0.71
	*Median*	0.10
	*Mean*	0.14
	*Standard deviation*	0.11
**Total wealth (£)**	*Minimum*	−1,578,980.00
	*Maximum*	20,818,008.00
	*Median*	200,600.00
	*Mean*	305,824.55
	*Standard deviation*	582,780.31
**Total annual income (£)**	*Minimum*	−10,000.00
	*Maximum*	507,645.94
	*Median*	19,866.00
	*Mean*	25,462.80
	*Standard deviation*	24,042.60

*Notes:* Baseline defined as respondent’s entrance into the ELSA study. Age values capped at 90 to prevent disclosure. Wealth and income measured at the benefit-unit level. Negative wealth values signify debt. Negative income values are possible, for example due to self-employed individuals whose business has made a net loss.

**Table 3:** Coefficients for Models 2–4

	Model 2		Model 3		Model 4	
	Beta	95% CI	Beta	95% CI	Beta	95% CI
**Within-effects**						
Age	0.037***	0.036, 0.039	0.037***	0.036, 0.039	0.037***	0.036, 0.039
Age^2^	0.012***	0.011, 0.013	0.011***	0.010, 0.012	0.012***	0.011, 0.013
LLPI			0.012***	0.011, 0.014		
Wealth percentile					−0.004***	−0.005, − 0.002
**Between-effects**						
Age	0.033***	0.031, 0.034	0.021***	0.019, 0.022	0.030***	0.028, 0.031
Age^2^	0.005***	0.003, 0.007	−0.001	−0.003, 0.000	0.002	−0.000, 0.003
LLPI			0.068***	0.066, 0.070		
Wealth percentile					−0.037***	−0.039, − 0.036
Sex (ref.: *Male)*						
*Female*	0.019***	0.016, 0.022	0.004**	0.001, 0.007	0.013***	0.010, 0.016
Education (ref.: *Less than upper secondary)*						
*Upper secondary/vocational*					−0.023***	−0.028, − 0.019
*Tertiary*					−0.031***	−0.036, − 0.026
Intercept	0.136***	0.133, 0.139	0.144***	0.142, 0.147	0.157***	0.153, 0.161
Within-R^2^	0.134		0.147		0.134	
Between-R^2^	0.109		0.426		0.251	
Overall R^2^	0.097		0.393		0.228	
N observations	62,694		62,694		57,453	
N individuals	15,733		15,733		14,349	

*** p < 0.001

** p < 0.01

* p < 0.05.
